# Mechanisms of Persistent Neurobiological Changes Following Adolescent Alcohol Exposure: NADIA Consortium Findings

**DOI:** 10.1111/acer.14154

**Published:** 2019-08-14

**Authors:** Fulton T. Crews, Donita L. Robinson, L. Judson Chandler, Cindy L. Ehlers, Patrick J. Mulholland, Subhash C. Pandey, Zachary A. Rodd, Linda P. Spear, H. Scott Swartzwelder, Ryan P. Vetreno

**Affiliations:** ^1^ Bowles Center for Alcohol Studies School of Medicine University of North Carolina Chapel Hill North Carolina; ^2^ Department of Neuroscience Charleston Alcohol Research Center Charleston South Carolina; ^3^ Department of Neurosciences The Scripps Research Institute La Jolla California; ^4^ Center for Alcohol Research in Epigenetics Department of Psychiatry University of Illinois at Chicago and Jesse Brown VA Medical Center Chicago Illinois; ^5^ Department of Psychiatry and Institute of Psychiatric Research Indiana University School of Medicine Indianapolis Indiana; ^6^ Developmental Exposure Alcohol Research Center Behavioral Neuroscience Program Department of Psychology Binghamton University Binghamton New York; ^7^ Department of Psychiatry and Behavioral Sciences Duke University Medical Center Durham North Carolina

**Keywords:** Adolescence, Binge Drinking, Development, Neuroimmune, Epigenetic, Behavior, Acetylcholine

## Abstract

The Neurobiology of Adolescent Drinking in Adulthood (NADIA) Consortium has focused on the impact of adolescent binge drinking on brain development, particularly on effects that persist into adulthood. Adolescent binge drinking is common, and while many factors contribute to human brain development and alcohol use during adolescence, animal models are critical for understanding the specific consequences of alcohol exposure during this developmental period and the underlying mechanisms. Using adolescent intermittent ethanol (AIE) exposure models, NADIA investigators identified long‐lasting AIE‐induced changes in adult behavior that are consistent with observations in humans, such as increased alcohol drinking, increased anxiety (particularly social anxiety), increased impulsivity, reduced behavioral flexibility, impaired memory, disrupted sleep, and altered responses to alcohol. These behavioral changes are associated with multiple molecular, cellular, and physiological alterations in the brain that persist long after AIE exposure. At the molecular level, AIE results in long‐lasting changes in neuroimmune/trophic factor balance and epigenetic–microRNA (miRNA) signaling across glia and neurons. At the cellular level, AIE history is associated in adulthood with reduced expression of cholinergic, serotonergic, and dopaminergic neuron markers, attenuated cortical thickness, decreased neurogenesis, and altered dendritic spine and glial morphology. This constellation of molecular and cellular adaptations to AIE likely contributes to observed alterations in neurophysiology, measured by synaptic physiology, EEG patterns, and functional connectivity. Many of these AIE‐induced brain changes replicate findings seen in postmortem brains of humans with alcohol use disorder (AUD). NADIA researchers are now elucidating mechanisms of these adaptations. Emerging data demonstrate that exercise, antiinflammatory drugs, anticholinesterases, histone deacetylase inhibitors, and other pharmacological compounds are able to prevent (administered during AIE) and/or reverse (given after AIE) AIE‐induced pathology in adulthood. These studies support hypotheses that adolescent binge drinking increases risk of adult hazardous drinking and influences brain development, and may provide insight into novel therapeutic targets for AIE‐induced neuropathology and AUDs.

Adolescent binge drinking remains a significant public health issue in the United States. In 2017, more than 4.5 million youth in the United States aged 12 to 20 (11.7%) reported binge drinking (4 + drinks for women, 5 + drinks for men), and almost 1 million (2.7%) reported frequent binge drinking (5 + binge drinking days in the past month) (SAMHSA, 2018). Extreme binge drinking of 10 to 15 or more drinks in a row is reported by many adolescents (Nguyen‐Louie et al., 2016; Patrick and Terry‐McElrath, 2019). It is clear that the short‐term consequences of adolescent binge drinking can be detrimental (Kuntsche and Gmel, 2013; White and Hingson, 2014), including effects such as impaired judgment, intoxicated driving, unintended sex, injury, and death. What is less clear is how binge drinking affects brain development, and whether such neurobiological effects resolve with time or persist into adulthood. To address the *causes* of excessive adolescent drinking, the National Institutes of Health has funded longitudinal research in youth to track which factors may predispose adolescents to binge drink. The National Consortium on Alcohol and Neurodevelopment in Adolescence (NCANDA) (Brown et al., 2015) and the Adolescent Brain Cognitive Development study (ABCD) (Auchter et al., 2018) are 2 large, multisite projects that aim to pinpoint environmental, social, and other factors that lead to drinking and drug use in adolescents. However, factors that predict alcohol use in humans can confound determination of the *consequences* of binge drinking, another major aspect of the public health issue and a question that can be systematically examined by modeling binge exposure in rodents that controls alcohol exposure across development.

In 2011, the National Institute on Alcohol Abuse and Alcoholism funded the Neurobiology of Adolescent Drinking in Adulthood (NADIA) Consortium with the charge to use animal models to define the enduring effects of binge‐like adolescent alcohol exposure and explore the associated neurobiological mechanisms. The Consortium gathered basic neuroscientists with multidisciplinary expertise to explore the impact of adolescent binge alcohol on adult neurobiology and psychopathology using rodent models. Individual laboratories would conduct independent but coordinated experiments, building a body of data that in aggregate could support or refute hypotheses on adolescent alcohol exposure. To integrate the projects and the resulting data, the Consortium agreed to a set of procedural guidelines. First, all studies used rats, and NADIA recommended procedures to control early‐life environments before adolescence, to reduce unintended impacts on adult neurobiology, alcohol drinking, and other phenotypes (Chappell et al., 2013; Yorgason et al., 2016). Second, alcohol exposure targeted the rat adolescent period, involving early adolescence, puberty, and young adulthood (i.e., broadly rat postnatal days [P] 25 to 55). Third, the alcohol exposure procedures were designed to mimic the intermittent, high blood alcohol levels common among binge‐drinking adolescent humans (Kuntsche and Gmel, 2013). Fourth, testing occurred in adulthood, typically P70 or later, to focus on the long‐lasting consequences of adolescent intermittent ethanol (AIE) exposure. Assessments following maturation to stable adult characteristics reduce developmental confounds that muddle multilaboratory replication efforts. The goal of these guidelines was to increase replication and extension of findings across NADIA Consortium components as well as to other alcohol research laboratories.

The NADIA Consortium designed dependent measures to probe a wide variety of behavioral, physiological, cellular, and molecular endpoints. Behavioral outcomes in adulthood, such as cognition, affect, and alcohol drinking, were essential to provide face validity for the AIE models. Next, the Consortium focused attention on mechanistic studies that cannot be performed in humans, using sophisticated neurobiological measures to characterize neural systems altered by AIE exposure and evaluating potential approaches for prevention or reversal of effects. After a brief summary of the behavioral alterations observed in the NADIA studies after AIE, this review focuses on NADIA Consortium findings that detail enduring AIE‐induced neurobiological alterations at the molecular, cellular, and physiological levels that likely underlie these behavioral changes. The use of distinct but overlapping measures, coupled with broad standard procedures, has allowed the NADIA laboratories to generate converging data, provide cross‐validation, and extend findings across a variety of alcohol exposures. The adolescent brain appears to be particularly vulnerable to the effects of binge alcohol exposure as compared to the adult brain, according to comparisons of animals exposed to ethanol (EtOH) during adolescence versus adulthood (Broadwater et al., 2014; Centanni et al., 2014; Fleming et al., 2013; Li et al., 2013; McClory and Spear, 2014; Risher et al., 2013; Vetreno et al., 2014; White et al., 2000, 2002a). While these comparisons provided the foundation for the formation of the NADIA Consortium, the current mandate is to focus on persistent AIE effects on brain and behavior and their underlying mechanisms. Thus, while the specificity of AIE effects as compared to adult exposure provided the foundation for the NADIA Consortium, it is not a focus of this review. This review describes NADIA discoveries with particular emphasis on emerging mechanistic findings in specific brain circuitry underlying AIE‐induced adult psychopathology.

## Persistent AIE‐Induced Effects on Behavior

AIE exposure affects a variety of behavioral measures, as summarized below and more thoroughly documented in recent focused NADIA reviews (Crews et al., 2016; Pandey et al., 2017; Spear, 2015, 2016a,2016b, 2018; Spear and Swartzwelder, 2014; Varlinskaya and Spear, 2015).

### Alcohol Drinking

Alcohol consumption in rodents is known to vary by species, strain, method, and environment (Crabbe et al., 1999; Fritz and Boehm, 2016). Nevertheless, the majority of rodent studies, including those from the NADIA Consortium, report that AIE often, although not always (Moaddab et al., 2017; Nentwig et al., 2019; Toalston et al., 2015; Varlinskaya et al., 2017), promotes adult alcohol drinking in multiple rat strains (Alaux‐Cantin et al., 2013; Broadwater et al., 2013; Gass et al., 2014; Lee et al., 2017; Pandey et al., 2015; Pascual et al., 2009; Rodd‐Henricks et al., 2002; Toalston et al., 2015; Wille‐Bille et al., 2017). These studies use a variety of AIE exposures as well as alcohol consumption paradigms. For example, adult EtOH drinking assessed by 2‐bottle choice in home cage increased following AIE exposure via self‐administration of sweetened alcohol (Broadwater et al., 2013), via i.p. injection (Pandey et al., 2015), or via a combination of self‐administration and vapor exposure (Criado and Ehlers, 2013). Similarly, a combined vapor/self‐administration AIE exposure (intermittent pattern of vapor exposure via voluntary drinking 20% unsweetened alcohol in male and female Wistar rats P22 to 62) increased adult EtOH consumption in both sexes, with females drinking more than males (Amodeo et al., 2018). Further, individual rats that consumed more EtOH during adolescence (high responders) showed the largest AIE‐induced increases in alcohol drinking in adulthood (Amodeo et al., 2017). AIE drinking or vapor exposure also increases operant responding for EtOH and reduces extinction of EtOH self‐administration (Amodeo et al., 2017; Gass et al., 2014). Thus, across rat strains, laboratories, and routes of administration, AIE often increases adult alcohol drinking.

### Anxiety

Another well‐documented effect of AIE is heightened social anxiety in adulthood (Varlinskaya and Spear, 2015), measured via the social interaction test (File and Seth, 2003). This finding is specific to males (Dannenhoffer et al., 2018; Varlinskaya et al., 2014, 2017) and to EtOH exposure during early adolescence (P25 to 45) as compared to late adolescence (P45 to 60) (Varlinskaya et al., 2014). Enhanced anxiety‐like behavior in adulthood after AIE exposure (via i.p., vapor, i.g., and self‐administration) has also been reported in the elevated plus maze (Kokare et al., 2017; Kyzar et al., 2017; Pandey et al., 2015; Sakharkar et al., 2016), the light–dark box (Lee et al., 2017; Pandey et al., 2015; Sakharkar et al., 2016; Slawecki et al., 2004; Vetreno et al., 2016), the marble‐burying test (Lee et al., 2017), and the open‐field test (Coleman et al., 2014; Vetreno et al., 2014). However, these findings are not universal, perhaps due in part to the induction of disinhibition, which has been reported in adult animals after vapor and self‐administered AIE in several studies (Desikan et al., 2014; Ehlers et al., 2019, 2013a; Gass et al., 2014; Gilpin et al., 2012). Specifically, it is well known that the behavioral expression of anxiety and disinhibition can compete depending on the characteristics of the test situation (Ennaceur, 2014). Thus, evidence supports both AIE‐induced anxiety and disinhibition.

### Learning and Behavioral Inflexibility

Investigations of the potential effects of AIE on learning and decision making in adulthood have revealed highly specific cognitive effects. While initial instrumental learning is generally unaffected (Boutros et al., 2016; Gass et al., 2014; Mejia‐Toiber et al., 2014; Risher et al., 2013; Semenova, 2012), operant behavioral inefficiency has been observed after AIE (Miller et al., 2017), and performance is impaired on more complex operant tasks that involve a rule change such as extinction or set‐shifting (Gass et al., 2014) and on nuanced memory tasks such as spatial–temporal object recognition (Swartzwelder et al., 2015). Similar findings have been reported in spatially based tasks such as the Morris water maze or the Barnes maze: Initial learning is intact, but changing the goal location reveals AIE‐induced deficiencies (Acheson et al., 2013; Coleman et al., 2011, 2014; Vetreno and Crews, 2012; Vetreno et al., 2019). These findings are interesting and can be interpreted as a loss of executive function and behavioral efficiency (Crews et al., 2016). AIE also has been shown to increase risky choices (Boutros et al., 2014; Schindler et al., 2014, 2016), measured by instrumental responding for a preferred reward, even under conditions where the choice is suboptimal. Both self‐administered and intragastric AIE exposure can enhance conditioning to reward predictive cues in adulthood (Kruse et al., 2017; Madayag et al., 2017; McClory and Spear, 2014; Spoelder et al., 2015), as indicated by approach to the cue (sign‐tracking). Finally, vapor AIE exposure in female rats promoted habit‐based alcohol seeking, indicating a loss of behavioral flexibility (Barker et al., 2017). Together, these findings have led to the hypothesis that AIE promotes behavioral inflexibility: Behavioral choices are biased toward rewards and incentive cues, and behaviors perseverate despite changing circumstances.

### “Lock‐in” of an Adolescent Phenotype

Multiple NADIA laboratories have observed that for some dependent measures, AIE induces a persistence of adolescent‐typical behavior or brain function into adulthood. This has been described as a “lock‐in” of adolescent characteristics, as if the adolescent alcohol exposure interrupted or altered neurobehavioral developmental processes, resulting in the expression of adolescent characteristics in adulthood (see Spear and Swartzwelder, 2014, for a review). Examples of adolescent characteristics that have been shown to persist into adulthood after AIE include the induction of memory‐related synaptic plasticity (Risher et al., 2015a), local circuit inhibitory processes (Fleming et al., 2012, 2013), increased impulsivity/disinhibition (Desikan et al., 2014; Ehlers et al., 2013a), and the dynamics of fear conditioning (Broadwater and Spear, 2014). Interestingly, several adolescent‐typical responses to EtOH are observed in AIE‐exposed adults, including elevated EtOH consumption (e.g., Alaux‐Cantin et al., 2013), insensitivity to EtOH on event‐related potential EEG responses (Ehlers et al., 2014), insensitivity to EtOH‐induced motor impairment (White et al., 2002b), increased sensitivity to EtOH‐enhanced social behavior (Varlinskaya et al., 2014), and increased sensitivity to EtOH‐induced impairment of working memory (White et al., 2000). Importantly, those changes do not appear to be related to tolerance, but rather to enduring physiological, and possibly molecular, alterations.

Collectively, these behavioral and cognitive studies have shown long‐lasting alterations induced by AIE. A similar conclusion has been reached from a recent review of studies in humans and rodents examining predisposing factors that predict adolescent alcohol consumption and the cognitive, behavioral, and neurobiological consequences of adolescent alcohol exposure (Spear, 2018). The animal data, in particular, support the hypothesis that binge‐like alcohol exposure has long‐lasting effects on behavior that may interact with, but are not dependent on, factors that predispose to adolescent drinking.

Conclusion: AIE exposure is sufficient to produce many behavioral characteristics (anxiety, behavioral inflexibility, increased drinking, and altered response to alcohol) observed in humans with alcohol use disorder (AUD). Additional studies are needed to clearly define the contribution of adolescent alcohol abuse to AUD.

## Persistent Changes in Adult Molecular Neurobiology Following Aie

NADIA studies have found that AIE persistently changes neuroimmune, neurotrophic, and epigenetic gene regulation and that these are key mechanisms underlying the AIE effects on adult physiology and behavior (for reviews, see Crews et al., 2017a; Crews and Vetreno, 2016; Crews et al., 2016; Kyzar et al., 2016; Pandey et al., 2017). These mechanisms involving epigenetic regulation of gene expression and noncoding RNA, particularly microRNA, involve signaling across neurons, astrocytes, and microglia that shift transcription, with increases in transcription of proinflammatory genes and reduced transcription of trophic factors.

### Neuroimmune Signaling

**Figure 1:**
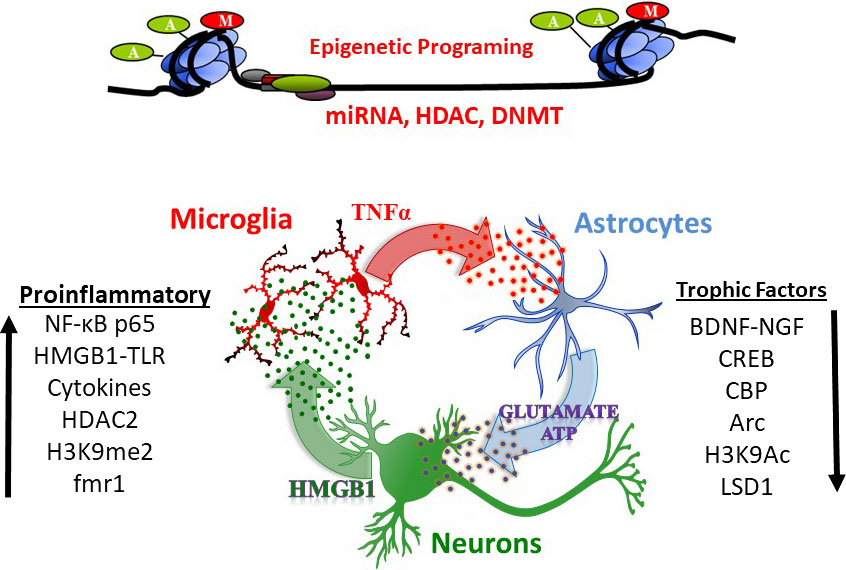
Molecular mechanisms of persistent changes in proinflammatory and trophic gene expression induced by adolescent intermittent ethanol (AIE) exposure. Top: Neurobiology of Adolescent Drinking in Adulthood (NADIA) findings support an overall hypothesis that complex mechanisms involving epigenetic and noncoding RNA, particularly microRNA (miRNA), contribute to a persistent increase in proinflammatory gene expression and a loss of trophic factor expression due to signaling across neurons and glia, which contribute to persistent changes in adulthood. AIE exposure changes multiple levels of molecular signaling in adulthood, including alterations in microRNA (miRNA) and epigenetic programing that involves DNA and nuclear histone methylation and acetylation processes involved in silencing or enhancing gene expression (HDAC: histone deacetylase; DNMT: DNA methyltransferase). Bottom: Adolescent development involves signaling across neurons, microglia, and astrocytes that regulate synaptic maturation and neurocircuitry. AIE exposure increases expression of proinflammatory genes and proteins (left), including nuclear factor kappa B (NFκB), high mobility group box 1 protein (HMGB1), Toll‐like receptors (TLRs), cytokines and chemokines, and many other genes. Epigenetic markers of gene silencing such as HDAC2 and H3K9me2 are also increased after AIE. Other proteins, growth factors, and epigenetic markers are persistently increased or decreased after AIE exposure (right). Levels of the epigenetic marker H3K9ac, associated with increasing gene transcription, are reduced in brain by AIE in association with decreases in transcription of brain‐derived neurotrophic factor (BDNF) and nerve growth factor (NGF). AIE exposure reduces adult expression of CREB, CREB‐binding protein (CBP), and the immediate early gene Arc in amygdala. AIE persistently reduces lysine‐specific histone demethylase 1A (LSD1, also known as lysine‐specific demethylase 1A or KDM1A) that demethylates histone lysines to epigenetically regulate gene expression. These signaling mechanisms control gene expression in neurons, astrocytes, and microglia and can thereby alter synapses that change circuits. By targeting these signaling mechanisms, AIE exposure can produce long‐lasting impacts on neurocircuitry and neurobiology.

Several studies report that AIE increases adult neuroimmune signaling through HMGB1, Toll‐like receptors (TLRs), and proinflammatory chemokines and cytokines known to signal in the innate immune system through NFκB transcription mechanisms (Fig. 1). Moreover, similar increases in gene expression were observed in postmortem brains of humans with AUD (Crews et al., 2017b). While the persistent proinflammatory gene induction by AIE was initially surprising, neuroimmune genes and microglia are known to contribute to brain development at multiple levels (Brenhouse and Schwarz, 2016; Cowan and Petri, 2018; Lenz and Nelson, 2018). Comparisons of adolescent and adult neuroimmune responses to EtOH and endotoxin (i.e., lipopolysaccharide) suggest that adolescents may have a blunted neuroimmune response (Doremus‐Fitzwater et al., 2015). Low initial innate immune responses, like those in adolescence, are associated with modest but persistent increases in proinflammatory cytokines that sensitize to repeated exposure, a potential mechanism of the allostatic changes associated with repeated alcohol exposure (Coleman and Crews, 2018; Crews et al., 2017b). In contrast, large proinflammatory responses, similar to adult responses, trigger initial proinflammatory gene increases that in time shift to increases in other cytokines often associated with wound healing, loss of proinflammatory signals, and desensitized responses (López‐Collazo and del Fresno, 2013; Morris et al., 2014).

### Epigenetic Mechanisms

Neuroimmune signaling among microglia, astrocytes, and neurons regulates epigenetic enzymes that open or silence chromatin architecture leading to altered gene expression. Further, neuroimmune signals contribute to brain development through processes such as axon guidance and activity‐dependent synapse development (Boulanger, 2009) that are mediated in part through changes in DNA methylation and histone methylation/acetylation that regulate neurotrophic and neuroimmune gene expression during adolescent brain development (Kyzar et al., 2016). For example, AIE exposure, but not identical adult EtOH exposure, causes a persistent loss of adult hippocampal dentate gyrus neurogenesis that is associated with increased neuroimmune gene expression (Broadwater et al., 2014). AIE decreases histone acetylation and decreases adult hippocampal brain‐derived neurotropic factor (BDNF), a key trophic factor regulating neurogenesis (Sakharkar et al., 2016; Vetreno and Crews, 2018). Moreover, AIE‐induced decreases in histone acetylation (H3K9/14) of the BDNF gene promotor and reductions in neurogenesis markers in the hippocampus were normalized by treatment with HDAC inhibitors in adulthood. AIE‐induced histone modifications have been extensively studied in amygdala, where AIE increases HDAC2, a deacetylase that reduces histone acetylation and decreases expression of BDNF (particularly Exon IV), activity‐regulated cytoskeleton‐associated (Arc) protein, and dendritic spine density (Pandey et al., 2015). Interestingly, reductions in BDNF expression in the amygdala are also regulated by increases in global and BDNF gene‐specific H3K9me2 by decreases in lysine demethylases (LSD1) and the neuron‐specific LSD1 + 8a splice variant in adult rat amygdala after AIE (Kyzar et al., 2017). Increased melanocortin and decreased neuropeptide Y activity due to altered histone acetylation in the amygdala may also be important in AIE‐induced anxiety phenotypes in adulthood (Kokare et al., 2017). HDACs and histone acetyltransferases (HATs) interact and regulate histone acetylation mechanisms (Krishnan et al., 2014), and CREB‐binding protein (CBP) and p300 serve as HATs and regulate histone acetylation. AIE decreases CREB and CBP/p300 levels in the rat adult amygdala via decreases in histone acetylation levels at their promoters (Zhang et al., 2018). Thus, the persistent AIE‐induced decrease in adult neurogenesis is linked to epigenetic histone repression of BDNF as well as to persistent increases in neuroimmune gene signaling, and AIE‐induced condensed chromatin architecture in the amygdala may be related to increases in HDAC2, decreases in HATs and LSD1, and regulation of the expression of BDNF, Arc, and neuropeptide Y. AIE epigenetic modifications in amygdala gene expression are related to AIE‐increased alcohol drinking and anxiety (Kokare et al., 2017; Kyzar et al., 2017, 2019a; Pandey et al., 2015; Zhang et al., 2018), whereas hippocampal and cholinergic neuron AIE alterations in gene expression have been linked to changes in cognition, synapses, and synaptic physiology (Mulholland et al., 2018; Risher et al., 2015a,2015b; Swartzwelder et al., 2015; Vetreno et al., 2019).

### MicroRNA (miRNA)

Small miRNA are released in vesicles and signal across cells regulating synaptic plasticity (Cohen et al., 2011). In addition to miRNA involvement in gene expression by targeting mRNA stability, miRNA and other noncoding RNA participate in neuroimmune signaling through TLRs (Coleman et al., 2017; Crews et al., 2017b). Studies performed in adult amygdala reveal that miRNA‐494 interacts with CREB transcription factors and CBP/p300 to regulate anxiety‐like behaviors (Teppen et al., 2016). Moreover, an antagomir (miRNA blocker) of miRNA‐494 injected into central amygdala increases CBP/p300 and histone acetylation and provokes anxiolytic effects similar to acute EtOH exposure in rats (Kyzar et al., 2019b; Teppen et al., 2016). Another miRNA‐mediated mechanism is the recently discovered activation of TLR7 by miRNA let7. Levels of TLR7 are increased by alcohol exposure contributing to neuroimmune activation (Coleman et al., 2017) and are persistently elevated in adults following AIE (Crews et al., 2017b). Thus, AIE‐induced changes in gene expression appear to involve multiple complex epigenetic mechanisms involving alterations in proinflammatory and trophic factors, as well as genes involved in remodeling synapses and neurocircuitry.

Conclusion: AIE exposure induces long‐lasting, persistent increases in proinflammatory neuroimmune genes as well as epigenetic histone‐ and DNA‐modifying enzymes and miRNA. These alterations in turn may contribute to decreased trophic factor gene expression that impacts the adult brain synaptic transcriptome.

## Aie‐Induced Changes in Adult Brain Cell and Neuroanatomy

NADIA studies have found that AIE exposure changes adult brain regional gray and white matter, brain neurogenesis, cholinergic, dopaminergic, and serotonergic phenotypic markers, dendritic morphology, microglia, astrocytes, and expression of various peptide transmitters.

### Neuronal Markers

NADIA investigators have used multiple routes of EtOH administration and rat strains to demonstrate that AIE persistently decreases choline acetyltransferase immunoreactivity (ChAT+IR) in forebrain cholinergic neurons. The AIE‐induced reduction in forebrain cholinergic neurons has been observed in both rats and mice (Coleman et al., 2011; Ehlers et al., 2019; Zhang et al., 2018), is not found with identical adult exposure (Vetreno et al., 2014), is comparable in both males and females (Vetreno and Crews, 2018), and persists following AIE from P56 (young adults) to at least P220 (middle‐age adults) (Vetreno and Crews, 2018). The 20 to 30% decrease in ChAT+IR by AIE is associated with loss of muscarinic and nicotinic cholinergic receptor mRNA (Coleman et al., 2011) and vesicular acetylcholine transporter as well as long‐lasting cognitive flexibility deficits (Coleman et al., 2011; Vetreno and Crews, 2015, 2018). Dopamine‐related proteins, such as tyrosine hydroxylase and catechol‐O‐methyltransferase, are also reduced by AIE (Boutros et al., 2014; Trantham‐Davidson et al., 2017). Although less well explored, AIE reduces serotonergic markers as indicated by a loss of both serotonin and tryptophan hydroxylase 2 (Vetreno et al., 2017a). These AIE‐induced changes in phenotypic neuronal markers may reflect alterations in neurotransmission, which could underlie some of the persistent physiological and behavioral changes found in adults following AIE exposure.

### Glia and Glial–Neuronal Interactions

**Figure 2:**
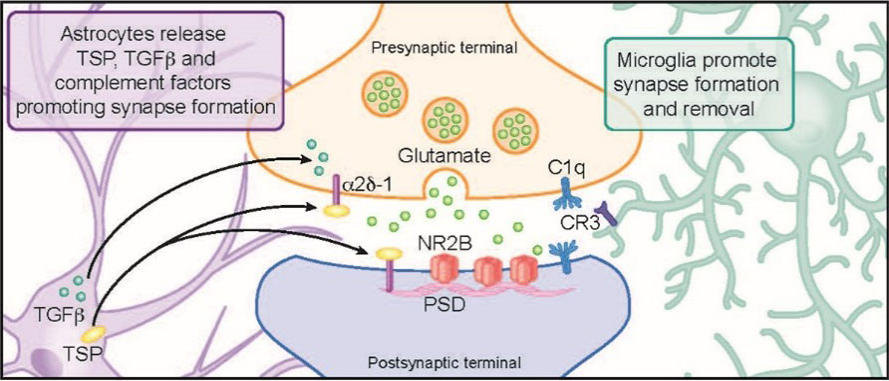
Glial and neuronal signals across neurons, astrocytes, and microglia lead to long‐lasting changes in synapses. Adolescent brain development occurs in part through maturation of neurocircuits and synapses. Synaptic development is regulated through astroglia and microglia. Thrombospondins (TSP) are signaling proteins released by astrocytes that promote synaptogenesis and are increased in adult hippocampus following adolescent intermittent ethanol (AIE; Risher et al., 2015b). Other studies find AIE alters multiple adult synaptic proteins, particularly the adult hippocampal excitatory synaptic NMDA receptor subtype GluN2B (NR2B) proteome (Swartzwelder et al., 2016) consistent with increases in synaptic excitation. Astrocytes stimulate the formation of synapses, and AIE has been found to increase astrocytic volume and area as well as increasing astrocyte‐secreted synaptogenic factors, thrombospondins (TSP), and their neuronal synaptic receptor alpha2delta‐1 (α2δ‐1). AIE also lowers adult hippocampal CA1 synaptic long‐term potentiation threshold, that is, increased potentiation, suggesting increases in excitatory synapses and/or NMDA (NR2B) receptors within excitatory synapses through association with the postsynaptic density 95 (PSD95) protein, a marker of excitatory synapses (Risher et al., 2015a). AIE increases the amplitude of NMDAR‐mediated currents in CA1 pyramidal cells and the proportion of that current driven by the NMDA excitatory receptor subunit containing GluN2B receptors (NR2B) (Swartzwelder et al., 2017). These findings suggest greater adult hippocampal network excitability after AIE, which alters plasticity mechanisms, like long‐term potentiation (LTP), and renders hippocampal circuits more vulnerable neurotoxic cell loss (Risher et al., 2015b). Similarly, AIE has been found to alter microglia, inducing a hyperramified state. Microglia regulate synapses in part through complement proteins, particularly C1q: complement component 1q, a protein complex that interacts with complement receptor 3 (CR3) (Walter and Crews, 2017). AIE increases adult microglial sensitivity to stimulation. Additional studies are needed to better understand how AIE‐induced persistent sensitization of microglia impacts synaptic regulation. Figure definitions: α2δ‐1: neuronal calcium channel subunit and binding site for thrombospondins; C1q: complement component 1q, a protein complex involved in the complement system of the innate immune system; CR3: phagocytotic complement receptor 3 of the innate immune system; NR2B: subunit of the NMDA receptor; PSD: postsynaptic density; TGFβ: transforming growth factor‐beta; TSP: thrombospondins

Emerging research shows that neurocircuitry is regulated in part through glial–neuronal signaling, with microglia, astrocytes, oligodendrocytes, and neurons all contributing (e.g., Fig. 1). For example, astroglia surround synapses, regulate synaptic transmission, and signal synapse formation, while microglia initiate and remove neuronal synapses (Fig. 2). Microglial markers are increased in postmortem brains of humans with AUD (He and Crews, 2008) and in adult rat brain following AIE (Sanchez‐Alavez et al., 2018a; Walter et al., 2017). However, these AUD‐ and AIE‐induced changes in microglia are subtle, unlike the dramatic changes following stroke or Alzheimer's disease (Crews et al., 2017a). AIE produces adult increases in hyperramified microglia morphology characterized by increases in protrusions and proinflammatory cytokine–chemokine secretion. Acute EtOH exposure increases expression of multiple innate‐immune signaling molecules in both neurons and microglia (Lawrimore and Crews, 2017), possibly indicating environment‐induced plasticity (Netea et al., 2015, 2016). Interestingly, AIE primes microglia responses to stress in prefrontal cortex (PFC) and other brain regions, whereas brain microglial depletion blocks some but not all EtOH‐stimulated proinflammatory genes, consistent with neurons and other glia contributing to these in vivo responses (Walter and Crews, 2017). It is unknown whether this priming is specific to AIE or whether similar effects would occur in response to adult alcohol exposure. Similarly, astrocyte activity is altered by AIE. Recent NADIA discoveries show that AIE increased GFAP staining, astrocyte‐released thrombospondins 2 and 4, and the neuronal α2δ‐1 calcium channel subunit with which they interact in adult hippocampal CA1 (Risher et al., 2015b) (Fig. 2). This suggests AIE‐induced excitatory synaptogenesis in adulthood, consistent with NADIA findings of reduced threshold for long‐term potentiation (LTP) (Risher et al., 2015a) and increased NMDA synaptic currents (Swartzwelder et al., 2017). Thus, signaling across glia and neurons is altered by AIE, consistent with the induction of synaptic changes in multiple brain regions.

### Dendritic Spines

NADIA studies have found AIE alters adult dendritic spine density and shifts spine subtypes in multiple brain regions (Mulholland et al., 2018; Risher et al., 2015a; Trantham‐Davidson et al., 2017), a finding that has recently been extended to mice (Jury et al., 2017). Dendritic spine changes are brain region–specific; for example, in the central and medial, but not basolateral amygdala, AIE exposure reduces total dendritic spine density (Jury et al., 2017; Pandey et al., 2015), whereas in medial PFC, AIE increases spine density (Trantham‐Davidson et al., 2017). The functional status of decreased dendritic spines in the amygdala in adulthood after AIE is not known. However, we recently demonstrated that AIE reduces several synaptic markers in the amygdala in adulthood (Kyzar et al., 2019a). For example, expression of synaptic plasticity–associated genes (BDNF, Arc, synaptophysin, NeuroD1, and NeuroD2) was decreased in the amygdala after AIE in adulthood. Furthermore, synaptophysin labeling (a marker of synapses) was decreased in the central and medial amygdala after AIE in adulthood (Kyzar et al., 2019a). These recent findings combined with decreased dendritic spines in the amygdala (Pandey et al., 2015) suggest that AIE produces altered synaptic function in the amygdala after AIE in adulthood. Thus, the persistence of AIE‐induced changes in dendrites and synapses likely reflects altered adult neurocircuitry.

### Neurogenesis

Neurogenesis, the formation of new neurons, continues in rat brain hippocampus and subventricular zone throughout adolescence into young adulthood, providing an index of brain plasticity. In adults, EtOH exposure reduces hippocampal neurogenesis; however, abstinence results in restoration after several weeks (Crews and Nixon, 2009). In contrast, adolescents have high levels of hippocampal neurogenesis that are more sensitive to EtOH inhibition than adults. NADIA studies across laboratories, using multiple routes of EtOH administration and rat strains, find that AIE persistently decreases neurogenesis in both the hippocampus and subventricular zones (Broadwater et al., 2014; Sakharkar et al., 2016; Vetreno and Crews, 2015). Moreover, AIE‐induced loss of hippocampal neurogenesis is significantly associated with increases in disinhibitory behavior (Ehlers et al., 2013a). The AIE‐induced persistent loss of neurogenesis likely reflects the AIE‐increased proinflammatory and reduced trophic factor expression (Fig. 1) in the hippocampus (see prevention‐reversal studies below).

### Whole‐Brain Structure

Several NADIA studies have characterized rat brain development and AIE effects using MRI to correspond to the extensive MRI literature on human brain. Rat brain MRI methods development indicated large age‐related changes in brain regional volume across puberty continuing into adulthood. Similar to humans, rat brain white matter grows throughout adolescence, contributing to changes in cortical thickness; however, one difference is that male rat brain continues to grow in adulthood. NADIA MRI studies found that AIE reduces adult hippocampal and white matter volume while increasing ventricular volumes, consistent with findings in humans with AUD (Ehlers et al., 2013b; Gass et al., 2014; Jacobus and Tapert, 2013). Diffusion tensor imaging (DTI) anisotropy provides both white and gray matter integrity information, and AIE alterations in DTI hippocampal anisotropy correlate with cognitive dysfunction (Vetreno et al., 2016). Cortical thickness changes across adolescence in humans (Ehlers et al., 2013b; Gass et al., 2014), and NADIA studies found that rat cortical thickness also changes with age, with AIE altering adult cortical thickness in multiple brain regions, including the insula, a brain region linked to alcohol exposure (Vetreno et al., 2017b). Notably, the AIE‐induced changes in cortical thickness detected with MRI were confirmed in histological determinations of postmortem rat brain, something rarely possible in human studies. Recently, the NADIA Consortium has applied resting‐state MRI of living animals to quantify AIE reductions in adult cortical‐striatal connectivity and responses to alcohol challenge (Broadwater et al., 2018). MRI connectivity studies are emerging as a convergent translational approach to integrate preclinical and clinical research.

Conclusion: AIE exposure induces enduring cellular and anatomical changes in adult brain, many of which have been observed in humans with AUD. These cellular and structural changes are likely to underlie many of the consequences on AIE exposure on neurocircuitry and behavior.

## Aie‐Induced Changes in Physiology

The NADIA Consortium has studied multiple physiological endpoints, including hippocampal synaptic physiology, PFC synaptic physiology, electroencephalogram (EEG) activity, sleep parameters, and resting‐state MRI brain connectivity, and documented several specific and persistent AIE‐induced effects (for reviews, see Crews et al., 2016; Ehlers and Criado, 2010; Spear and Swartzwelder, 2014).

### Synaptic Physiology

Several NADIA studies find alterations in synaptic physiology following AIE that support a shift to increased excitation (Figs 1 and 2), with hippocampal and prefrontal synapses being the most studied regions. Hippocampal extra‐synaptic GABA_A_ receptors (GABA_A_R) maintain a tonic inhibition in the dentate gyrus that is uniquely responsive to acute EtOH in adolescents compared to adults (Fleming et al., 2011). AIE, but not equivalent adult exposure, maintains an adolescent level of tonic current EtOH sensitivity into adulthood (Fleming et al., 2012, 2013) and also alters GABA_A_R subunit mRNA and protein expression (Centanni et al., 2014). These changes were also found to occur in layer V pyramidal neurons of the PFC, but not layer II/III neurons, in male and female animals (Centanni et al., 2017). In studies on *I*_A_ (peak *I*_A_ amplitude/membrane capacitance) and cell capacitance in hippocampal CA1 interneurons, AIE reduced *I*_A_ density, with no change after equivalent adult exposure (Li et al., 2013). Interestingly, AIE sensitizes adult CA1 LTP, suggesting a lower threshold for LTP induction (Risher et al., 2015a), consistent with altered increases in the amplitude of adult NMDAR‐mediated currents through GluN2B receptors (Swartzwelder et al., 2017). This increased excitability by AIE could underlie a neurotoxic loss of CA1 neurons, as indicated by reduced NeuN staining (Risher et al., 2015b). These synaptic findings are consistent with AIE‐induced changes in adult hippocampus that perpetuate adolescent‐like physiological function and response to alcohol into adulthood (see Spear and Swartzwelder, 2014, for a review).

### Dopamine and Decision Making

Dopaminergic neurotransmission in the PFC modulates cognitive function and changes dramatically during adolescence. AIE reduced markers of dopamine innervation and revealed a loss of dopamine D1 receptor modulation of evoked firing and NMDA currents without altering dopamine D2 receptor function (Trantham‐Davidson et al., 2017). This differential effect of AIE on D1 and D2 function is in direct contrast to the effect of chronic alcohol exposure in adult animals, in which a loss of prelimbic D2 receptor function was observed without a change in D1 receptor function (Trantham‐Davidson et al., 2014). As mentioned earlier, PFC executive functions are compromised by AIE. Multiple AIE studies have examined risky decision making following adolescent alcohol exposure via gavage or self‐administration (Boutros et al., 2014; Schindler et al., 2014, 2016). Risky‐choice behaviors assessed using a probability discounting task indicated that adult AIE‐exposed rats exhibited an increase in risky‐alternative responses that resulted in fewer rewards (Boutros et al., 2014). This study reported AIE‐induced reductions in both adult PFC tyrosine hydroxylase and ChAT, although PFC ChAT was correlated with increased risky decisions. Another study (Schindler et al., 2016) found that self‐administered AIE altered mesolimbic dopamine signaling in adulthood via the cholinergic input from the pedunculopontine tegmentum to the ventral tegmental area, while comparable adult EtOH exposure did not. Further, the altered dopamine signaling following AIE was correlated with an increase in risky‐choice behaviors and could be reversed by treatment with a selective GABA_A_ allosteric agonist (Schindler et al., 2016). In addition to risky decisions, AIE‐enhanced mesolimbic dopamine has been linked to increased adult voluntary EtOH drinking in alcohol‐preferring (P) rats related to sensitization to EtOH reinforcement (Toalston et al., 2014). Moreover, increased adult self‐administration of EtOH following AIE may be associated with altered effects of adult EtOH challenge on dopamine release, as reported in anesthetized rats (Shnitko et al., 2016). Together, these studies suggest that synaptic and circuit changes following AIE are likely to underlie observed changes in decision making and alcohol drinking.

### EEG, Sleep, and Resting‐State Connectivity

NADIA studies found that AIE exposure can lead to deficits in a number of electrophysiological and behavioral responses in adulthood that extend mechanistic studies and translate to humans. For instance, AIE reduced EEG prepulse inhibition of startle response, and that reduction was significantly associated with reduced hippocampal size (Ehlers et al., 2013b). AIE also diminished the amplitude and timing of slow‐wave sleep in adulthood (Criado et al., 2008; Ehlers et al., 2018). This is consistent with human research, as reductions in slow‐wave sleep are present in adult humans with AUD (e.g., Irwin et al., 2000), and young adults with a history of heavy binge drinking during adolescence exhibit poor sleep quality (Ehlers et al., 2018). NADIA researchers also investigated phase‐locking of event‐related oscillations (EROs) between cortical sites. EROs in humans may be biomarkers of AUD or endophenotypic markers of the vulnerability to AUD (Rangaswamy et al., 2007). Ehlers and colleagues found that a history of vapor AIE was also associated with lower levels of ERO phase‐locking between frontal and parietal cortex in the theta frequencies of AIE rats when compared to controls (Ehlers et al., 2018; Sanchez‐Alavez et al., 2018b), a finding also seen in young adult humans with a history of heavy binge drinking (Ehlers et al., 2019). Further, cholinergic lesions in medial septum alter ERO phase synchronization between cortex and other brain regions, while cholinergic lesions in nucleus basalis magnocellularis modify synchronization/phase resetting within the region (Sanchez‐Alavez et al., 2014), consistent with AIE reducing both ERO synchronization and ChAT + IR neurons in these cholinergic nuclei. In adult humans, resting low‐voltage alpha EEG is heritable and associates with aspects of binge drinking and a less intense response to alcohol (Ehlers et al., 2015). The electrophysiological endpoints represent opportunities for translation of NADIA findings to human studies (Ehlers et al., 2019).

Consistent with the electrophysiological desynchronization after AIE, NADIA studies discovered that AIE decreased resting‐state MRI connectivity among PFC subregions as well as between PFC and striatal regions (Broadwater et al., 2018). The findings in animals agree with human studies that reported decreased resting‐state connectivity in adult AUD (Weiland et al., 2014) and in high‐risk young adults with a family history of AUD (Weiland et al., 2013). Interestingly, Broadwater and colleagues (2018) also found that AIE blunted the acute connectivity response to alcohol challenge, further supporting other findings of AIE‐induced changes in adult responses to alcohol. The AIE‐induced loss of cortical connectivity in adulthood is consistent with disruption of decision making and increased risky decisions. Future functional connectivity studies are expected to integrate rat AIE findings of the NADIA Consortium to emerging studies on human adolescence, providing critical links to molecular mechanisms that translate to humans.

Conclusion: AIE induces long‐lasting changes in adult brain physiology. Hippocampal synaptic markers, as well as GABA_A_Rs, potassium channels, and LTP, are modified. PFC and mesolimbic circuit changes in adults after AIE are linked to cognitive deficits such as enhanced risk‐taking. AIE‐induced changes in adult EEG, ERO, sleep, and functional connectivity suggest disrupted brain regional interaction that may represent blunted development of cortical circuits.

## Prevention and Reversal of AIE Consequences

**Figure 3:**
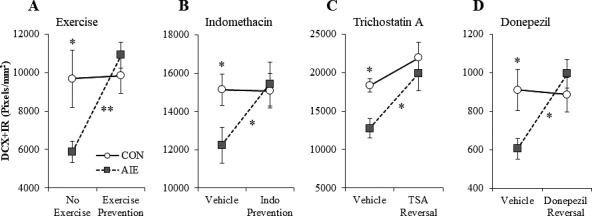
Adolescent intermittent ethanol (AIE)‐induced reductions in adult neurogenesis are prevented or reversed by multiple strategies. Doublecortin immunoreactivity‐positive (DCX+IR) cell counts are adapted from Neurobiology of Adolescent Drinking in Adulthood (NADIA) publications across multiple NADIA laboratories studying the impact of AIE on adult neurogenesis. Note different ordinate scales, as pixel densities are influenced by within‐experiment differences in chromagen color, antibodies, and signal‐to‐background ratios. (**A**) Vetreno and colleagues (2018) exposed rats to AIE (postnatal day [P] 25 to 55, i.g.) and assessed DCX+IR neurogenesis at P80. Voluntary wheel running from P24 to P80, concurrent with and extending beyond AIE exposure, prevented the AIE‐induced loss of DCX+IR as well as other markers of neurogenesis without significantly altering neurogenesis in control rats. Exercise also prevented AIE induction of pNFκB p65 and proinflammatory gene mRNA. (**B**) Vetreno and colleagues (2018) exposed rats to AIE (P25 to 55, i.g.) and assessed DCX+IR neurogenesis at P56, shortly after AIE. Indomethacin, an antiinflammatory drug, was administered during AIE (4 mg/kg, i.p.) and prevented the AIE‐induced loss of DCX+IR. Indomethacin also prevented AIE induction of pNFκB p65 and cleaved caspase‐3. (**C**) Sakharkar and colleagues (2016) exposed rats to AIE (P28 to 41, i.p.) and assessed DCX+IR neurogenesis at P94. Three days of trichostatin A (TSA; 1 mg/kg, i.p., P92 to 94) reversed the AIE reduction in DCX+IR. TSA also reversed AIE‐induced increases in histone deacetylase activity and reductions in brain‐derived neurotropic factor (BDNF) mRNA and H3K9ac levels of the BDNF promoter. (**D**) Swartzwelder et al. (2019) exposed rats to AIE (P30 to 46, i.g.) and assessed DCX+IR neurogenesis at P72. Donepezil, an anticholinesterase with antiinflammatory activity, was administered for 4 days (2.5 mg/kg, i.p., P67 to 72) and reversed AIE reductions in DCX+IR. Donepezil also reversed the AIE‐induced increases of RAGE, phosphorylated (activated) nuclear transcription factor pNFκB p65, and the gene silencing marker dimethylated histone H3K9.

NADIA studies have used targeted strategies focused on prevention and/or reversal of AIE‐induced changes in adults. The rationale is that prevention of AIE molecular and behavioral changes in adulthood tests mechanistic hypotheses of AIE, while reversal of already‐established AIE effects may reveal therapeutic targets for AUD. Several NADIA studies have reported prevention or reversal of AIE pathology using exercise, the antiinflammatory drug indomethacin, the anticholinesterase drug donepezil, the anticonvulsant gabapentin, and the histone deacetylase inhibitor trichostatin A (TSA). One consequence of AIE that has proven to be malleable is blunted neurogenesis (Fig. 3). Alterations in adult hippocampal neurogenesis have been linked to stress‐induced depression (Eisch and Petrik, 2012), impaired cognitive flexibility (Anacker and Hen, 2017), and age‐related cognitive decline (Apple et al., 2017), as well as alcohol withdrawal, neurodegeneration, and abstinence regeneration (Crews and Nixon, 2009). AIE uniquely causes a persistent loss of neurogenesis, unlike identical adult EtOH exposure (Broadwater et al., 2014), consistent with a permanent decrease in the stem cell pool. NADIA‐supported neurogenesis prevention and reversal studies have established that AIE shifts the proinflammatory/trophic gene expression balance through epigenetic mechanisms. Doublecortin immunostaining visualizes newly forming neuroprogenitors that are persistently decreased in AIE‐treated young adults across multiple studies and NADIA laboratories. Exercise (Vetreno et al., 2018), the antiinflammatory drug indomethacin (Vetreno et al., 2018), TSA (Sakharkar et al., 2016), and the anticholinesterase inhibitor donepezil ([Link]) all prevent and/or reverse the AIE‐induced loss of neurogenesis (Fig. 3). All of these treatments reduce proinflammatory gene expression, and many increase trophic gene expression through complex mechanisms. Donepezil may act directly on microglia (Hwang et al., 2010; Kim et al., 2014), reducing proinflammatory genes as well as potentiating acetylcholine inhibition of inflammation (Cutuli et al., 2013). Histone deacetylase inhibitors block endotoxin induction of brain proinflammatory genes and reverse the AIE‐induced persistent loss of hippocampal BDNF (Sakharkar et al., 2016). Donepezil also reverses AIE‐induced hippocampal dendritic spine adaptations and epigenetic induction of the *Fmr1* gene, which is linked to synaptogenesis (Mulholland et al., 2018). Most recently, subchronic donepezil treatment of adult rats has been shown to reverse AIE‐induced reduction of hippocampal neurogenesis, increases of cell‐death markers, and the increases of neuroimmune signaling molecules transcription factors and epigenetic marks that accompany those changes (Swartzwelder et al., 2019). Together these studies find that drug or exercise reversal of AIE‐induced increases in proinflammatory genes and reduced trophic gene expression can prevent and/or reverse AIE pathology.

**Figure 4:**
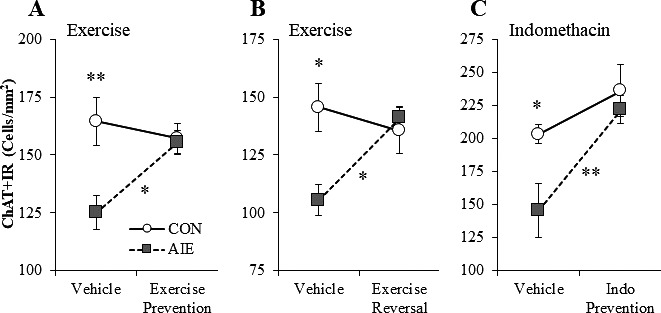
Adolescent intermittent ethanol (AIE)‐induced reductions in choline acetyltransferase (ChAT) are prevented or reversed by multiple strategies. ChAT immunoreactivity‐positive (DCX+IR) cell counts are adapted from Neurobiology of Adolescent Drinking in Adulthood (NADIA) publications studying the impact of AIE on cholinergic phenotype. Note different ordinate scales. (**A**) Vetreno and Crews (2018) exposed rats to AIE (postnatal day [P] 25 to 55, i.g.) and assessed ChAT+IR at P80. Voluntary wheel running from P24 to P80, concurrent with and extending beyond AIE exposure, prevented the AIE‐induced loss of ChAT+IR without significantly altering levels in control rats. (**B**) Vetreno and colleagues (2019) exposed rats to AIE (P25 to 55, i.g.) and assessed ChAT+IR at P95. Voluntary wheel running from P56 to P95, after AIE exposure, reversed the AIE‐induced loss of ChAT+IR without significantly altering levels in control rats. (**C**) Vetreno and Crews (2018) exposed rats to AIE (postnatal day [P] 25 to 55, i.g.) and assessed ChAT+IR at P56, shortly after AIE. Indomethacin, an antiinflammatory drug, was administered during AIE (4 mg/kg, i.p.) and prevented the AIE‐induced loss of ChAT+IR.

**Figure 5:**
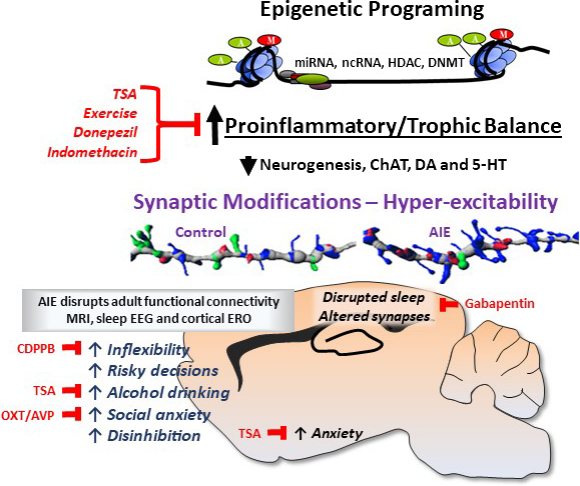
Prevention and reversal of adolescent intermittent ethanol (AIE) neuropathology by multiple strategies. Top: Epigenetic mechanisms involving histone and DNA methylation and acetylation are known mechanisms regulating gene expression, that is, silencing or enhancing transcription, and are persistently altered by AIE. Neurobiology of Adolescent Drinking in Adulthood (NADIA) findings support a shift in proinflammatory/trophic balance caused by AIE activation of neuroimmune signaling through increased high mobility group box 1 protein (HMGB1), Toll‐like receptors, and other signaling that increase nuclear factor kappa B (NFκB) transcription and blunt transcription of trophic factors such as brain‐derived neurotrophic factor (BDNF) and nerve growth factor (NGF). These changes likely lead to the observed AIE‐induced reductions in neurogenesis and neuronal phenotype markers (choline acetyl transferase [ChAT], dopamine [DA], serotonin [5‐HT]), as well as a shift toward synaptic excitability (middle). Together, these physiological changes may manifest as AIE alterations in a variety of neuronal circuit and behavioral effects (bottom), including disrupted sleep, anxiety, risky decisions, alcohol drinking, and cognitive inflexibility. NADIA AIE studies find that a variety of pharmacological and behavioral strategies (shown in red) can prevent or reverse these AIE effects. Exercise and the antiinflammatory drug indomethacin prevent AIE‐induced changes in neurogenesis, ChAT, and cognitive inflexibility (Vetreno et al., 2018). Donepezil, an anticholinesterase that is also antiinflammatory, reverses AIE‐induced changes in epigenetic programing of proinflammatory genes, trophic factors, histones, and synapses ([Link]). The histone deacetylase inhibitor trichostatin A (TSA) reverses AIE‐induced decreases in trophic factor expression, synapses, and neurogenesis as well as increased alcohol drinking and anxiety (Sakharkar et al., 2016). Other pharmacological agents including the positive allosteric modulator of type‐5 metabotropic glutamate receptor CDPPB (Gass et al., 2014), oxytocin and vasopressin receptor ligands (Dannenhoffer et al., 2018), and the anticonvulsant gabapentin (Sanchez‐Alavez et al., 2018b; Swartzwelder et al., 2017) have reversed specific behavioral effects. Although no individual study has integrated all potential mechanisms and level of assessment, overall the prevention and reversal studies together strongly support an AIE‐induced persistent shift in brain proinflammatory/trophic gene expression that crosses glia and neurons, altering synapses and neurotransmitters that contribute to long‐lasting changes in adult neurophysiology, neurocircuitry, and behavior.

The studies demonstrating reversal of a persistent AIE effect are surprising and exciting, although more studies are needed to integrate these prevention and reversal mechanisms. Exercise and indomethacin also prevent the AIE‐induced loss of cholinergic forebrain neurons (Fig. 4). Exercise is generally accepted as improving health through complex mechanisms that include increased trophic factor expression and reduced proinflammatory responses. Exercise and the antiinflammatory drug indomethacin prevent AIE‐induced loss of multiple cholinergic neuron markers (e.g., ChAT, the vesicular acetylcholine transporter, and the NGF receptor TrkA), as well as AIE‐induced increases in forebrain pNFκB, a transcription factor linked to proinflammatory gene induction (Vetreno and Crews, 2018). Cholinergic neurons are dependent upon NGF from target regions, and exercise and indomethacin prevent the AIE‐induced loss of TrkA and hippocampal NGF (Vetreno and Crews, 2018). AIE‐induced cognitive deficits are also reversed by exercise (Vetreno et al., 2019). These studies are consistent with the hypothesis that AIE‐associated loss of cholinergic neurons is due to increased proinflammatory cytokines and reduced trophic factors (i.e., both NGF and TrkA, its receptor on ChAT neurons), causing a shift in the proinflammatory/trophic balance critical for cholinergic phenotype. Reversal of the loss of cholinergic neurons by exercise was surprising, prompting experiments on mechanisms that led to the discovery of epigenetic silencing of cholinergic phenotypic genes. As AIE did not reduce forebrain neurons (indexed via NeuN) or induce basal forebrain neurogenesis (assessed via BrdU), the AIE reductions of cholinergic neurons appeared to involve a loss of the cholinergic phenotype and associated cognitive deficits that did not involve neuronal death. Instead, assessments of ChAT and TrkA DNA and histone markers suggested that proinflammatory pNFκB silences ChAT and TrkA gene expression, resulting in loss of the cholinergic phenotype while perhaps sparing the release of remaining neurotransmitters. Similar mechanisms may underlie many of the persistent AIE‐induced changes in synapses, glia, other neurons, circuits, and behavioral pathologies. Although few individual studies have simultaneously integrated these different mechanisms, together these findings strongly support the hypothesis that AIE persistently shifts brain proinflammatory/trophic gene expression across glia and neurons, altering synapses and neurotransmitters that contribute to long‐lasting changes in neurophysiology and neurocircuitry (Fig. 5).

The NADIA Consortium has also explored prevention and reversal of AIE‐induced behavioral changes. TSA administration reverses AIE‐increased adult alcohol drinking and anxiety‐like behavior as well as epigenetic and synaptic changes in amygdala (Kyzar et al., 2017; Pandey et al., 2015; Sakharkar et al., 2016). The anticonvulsant gabapentin reverses GluN2B receptor–mediated, AIE‐induced synaptic hyperexcitability (Swartzwelder et al., 2017; also see Swartzwelder et al., 2016). Gabapentin also increases slow‐wave sleep power, thereby reversing deficits seen in slow‐wave sleep in AIE‐exposed adults (Sanchez‐Alavez et al., 2018b). CDPPB, a positive allosteric modulator of type‐5 metabotropic glutamate receptors, reversed AIE‐induced behavioral inflexibility in an operant set‐shifting task, without altering control rat behavior (Gass et al., 2014). Another example is the male‐specific AIE enhancement of social anxiety, which is related to changes in hypothalamic oxytocin/vasopressin ratio and is reversed by oxytocin receptor agonists or vasopressin V1b receptor antagonists (Dannenhoffer et al., 2018). While fewer studies have focused on preventing AIE‐associated behavioral deficits, exercise during AIE prevents AIE‐induced cognitive inflexibility (Vetreno et al., 2018). NADIA AIE prevention and reversal experiments support the molecular mechanisms described above (Fig. 4), but more studies integrating multiple molecular endpoints to physiological and behavioral studies are needed to fully understand these mechanisms. The emerging molecular mechanisms provide potential targets for treatments of AUD, since many of the enduring changes in brain found in NADIA studies mimic those found in human AUD.

Conclusion: AIE‐induced molecular, synaptic, physiological, and behavioral changes can be prevented and/or reversed by blocking proinflammatory gene induction, inhibiting histone deacetylation, and/or by targeted pharmacological treatment.

## Discussion and Summary

The NADIA Consortium initially hypothesized that adolescent binge drinking can lead to persistent changes in adult neurobiology. By developing reliable AIE models, the Consortium has discovered multiple long‐lasting effects of AIE across a broad spectrum of molecular, synaptic, cellular, physiological, behavioral, and alcohol‐response endpoints. The persistence of AIE‐induced changes is surprising. Somewhat unexpectedly, NADIA investigators discovered that AIE is sufficient to enhance many adult behavioral characteristics that were previously thought to represent trait vulnerabilities, such as anxiety, behavioral inflexibility, risky decisions, altered response to alcohol, and increased alcohol consumption—characteristics observed in individuals with AUD. Moreover, some of the cellular and molecular consequences of AIE are translational and observed in postmortem brains of humans with AUD. This is consistent with human studies linking early‐onset drinking with increased risk for AUD. The discovery that the molecular proinflammatory/trophic gene balance and epigenetic mechanisms that were shown to be induced by AIE are long‐lasting and responsible for adult psychopathology strengthens the basis for prevention. Further, these newly discovered mechanisms are likely to emerge as major developmental plasticity processes modulated by alcohol and other environmental factors that impact adolescent development and adult neurobiology.

The AIE model of adolescent binge drinking followed by abstinence into adulthood was developed specifically to investigate the impact of adolescent binge exposure and is not focused on preclinical lifelong drinking models. Human adolescent alcohol abuse is difficult to model. Studies of drinking in 18‐ to 19‐year‐olds find large variation in amounts of drinking over time, including periods of no drinking as well as heavy binge drinking, dependent upon holidays, events, and other factors (Goldman et al., 2011). Preclinical models are impacted by differences in experimental design (e.g., route and timing of EtOH administration, specific assessments made) and also environmental factors (location, vivaria) that could contribute to variations in outcomes within and outside the Consortium. Moreover, while initial evidence suggests sex differences in some of these effects, the role of sex has yet to be explored in detail. The extent to which AIE model results and conclusions vary as a consequence of these differences is important but unknown; nevertheless, the relative consistency of AIE exposure on adult alcohol drinking, anxiety, disinhibition, and cognitive flexibility indicates that the alcohol exposure reliably impacts brain development. The NCANDA and ABCD longitudinal studies of human adolescents were designed to provide insight into individual psychological and physiological phenotypes associated with different drinking trajectories in both males and females, and the NADIA Consortium can use that information to design preclinical mechanistic studies. Assessment of individual trajectories of alcohol‐related problems is important. For example, a recent NADIA study found that heavier drinking adolescent rats also showed larger AIE‐induced increases in alcohol drinking in adulthood (Amodeo et al., 2017). How individual differences, including sex differences, impact the proinflammatory/trophic and epigenetic mechanisms that NADIA has identified will require additional investigation.

NADIA findings indicate that AIE changes brain regional gene expression, synapses, synaptic physiology, cortical thickness, and white matter, as well as altering cholinergic and other neuronal phenotypes. These changes are consistent with the physiological aspects of brain regional function that are persistently disrupted following AIE, such as ERO, sleep stages, EEG synchronization, and resting‐state MRI functional connectivity. Additional studies are needed to fully integrate the molecular and synaptic mechanisms and define the neural circuits that underlie the changes in physiology and behavior across cortical and subcortical brain regions. Recent studies find that molecular epigenetic modifiers of RNA and DNA can be inherited, including those that modify alcohol and stress responses (Chastain and Sarkar, 2017) that appear to underlie AIE molecular mechanisms. Currently, NADIA studies have established that AIE causes long‐lasting changes in adults that increase known risks for AUD as well as molecular changes in brain that mimic AUD pathology. Human and preclinical studies are converging upon endpoints to test the validity of these findings. NADIA discoveries support a role for adolescent epigenetic programming in the development of AUD; while inherited genetic factors also clearly contribute, additional studies are needed to better understand the how genetic and epigenetic factors influence adolescent developmental neurobiology and risks for AUD.

A major value of the AIE model is to investigate mechanisms underlying AIE impacts on neurobiology and behavior. In preclinical studies, exercise has been shown to increase trophic factor expression and blunt proinflammatory gene expression. The finding that exercise can block AIE‐induced proinflammatory, trophic, and epigenetic molecular changes, as well as cellular, physiological, and cognitive deficits, supports the hypothesis that disrupted neuroimmune and trophic gene expression are key mechanisms underlying persistent AIE‐induced changes in adult brain physiology, alcohol drinking, and alcohol response. The observations that the antiinflammatory indomethacin and the histone deacetylase inhibitor TSA can reverse some of the AIE effects support these hypothetical mechanisms, and suggest they are persistent but not permanent. This exciting discovery that at least some AIE‐induced alterations are reversible or modifiable provides motivation for prevention efforts and perhaps AUD therapeutic targets. The anticholinesterase donepezil both blunts microglial activation and reverses AIE‐induced pathology, likely acting in part through neuroimmune mechanisms. Thus, NADIA discoveries of unique adolescent programming of persistent changes in neurobiology through novel molecular mechanisms offer potentials for reversal via discovery of novel mechanisms and treatment targets for AUD.

In conclusion, NADIA mechanistic studies establish that adolescent binge drinking can induce long‐lasting changes in brain gene expression, synapses, electrophysiology, and morphology, as well as behavior. They have identified several tractable drug targets that can be useful in AUD treatment. Finally, these studies provide additional support for the public health importance of reducing and preventing underage drinking.

## Funding

The NADIA Consortium is supported by the NIH (U24AA020022, U24AA020024, U24AA024603, U24AA024605, U01AA019925, U01AA019967, U01AA019969, U01AA019970, U01AA019971, U01AA019972, U01AA019973, U01AA020023, U01AA024599, U01AA024612).

## Conflict of Interest

The authors declare no conflict of interest.
